# Open Distal Gastrectomy for Gastric Cancer in an Older Patient With Severe Chronic Obstructive Pulmonary Disease Under Combined Spinal–Epidural Anesthesia and Sedation: A Case Report

**DOI:** 10.1002/ccr3.71221

**Published:** 2025-10-14

**Authors:** Hiromi Shinohara, Gotaro Shirakami, Maki Ishii, Masahiro Kakuyama

**Affiliations:** ^1^ Department of Anesthesiology Kyoto City Hospital Kyoto Japan

**Keywords:** chronic obstructive pulmonary disease, combined spinal–epidural anesthesia, dexmedetomidine, gastrectomy, sedation

## Abstract

Only a few reports have described gastrectomy performed avoiding general anesthesia to prevent postoperative pulmonary complications in patients with severe respiratory dysfunction. An 80‐year‐old male patient with severe chronic obstructive pulmonary disease was scheduled to undergo surgery for advanced gastric cancer. We successfully managed him using combined spinal–epidural anesthesia with dexmedetomidine sedation during open distal gastrectomy.

**Summary**

- Combined spinal–epidural anesthesia with dexmedetomidine sedation can be an effective anesthetic management strategy for older patients with severe chronic obstructive pulmonary disease undergoing open distal gastrectomy for gastric cancer.

## 1 Introduction

Patients with chronic obstructive pulmonary disease (COPD) have increased risks of postoperative pulmonary complications (PPCs) following upper abdominal surgery [1, 2]. Moreover, general anesthesia (GA) is also widely recognized to be associated with an increased risk of PPCs in patients with COPD [3, 4]. Thus, minimizing GA use is crucial for the perioperative management of upper abdominal surgery in patients with COPD. Despite advances in anesthetic techniques, upper abdominal surgery without GA remains challenging for anesthesiologists. Furthermore, reports on upper abdominal surgery, particularly gastrectomy for gastric cancer, managed solely with regional anesthesia (RA), are extremely limited in patients with COPD. Here, we present a case of open distal gastrectomy for gastric cancer that was successfully managed under combined spinal–epidural anesthesia (CSEA) and sedation in an older patient with severe COPD.

## 2 Case History and Presentation

An 80‐year‐old man (weight: 46 kg; height: 160 cm) was scheduled for gastric cancer surgery for the treatment of stage III (T4N2M0) advanced cancer and had experienced severe difficulties with oral intake. The patient was an ex‐smoker of 60 cigarettes daily for 35 years until the age of 55. At the age of 75, the patient developed pneumothorax requiring chest drainage and was subsequently diagnosed with emphysema, leading to the initiation of inhaled bronchodilator therapy.

Preoperative pulmonary function tests indicated a one‐second forced expiratory volume (FEV1)/forced vital capacity (FVC) ratio of 33.1%. Chest computed tomography revealed an overinflated lung with numerous giant bullae. The surgeons determined that a distal gastrectomy or, as a palliative alternative, a gastrojejunostomy bypass procedure was necessary. They preferred to perform the procedure endoscopically under GA, if possible.

At our preoperative anesthesia clinic, the patient's COPD severity was classified as grade 3 according to the Global Initiative for Chronic Obstructive Lung Disease classification [5]. The dyspnea was classified as functional class 4 [6]. After discussions between the surgeons and anesthesiologists, open gastrectomy under CSEA was selected to avoid GA and maintain spontaneous breathing. The patient was informed that CSEA under sedation could reduce the risk of PPCs associated with GA. We also explained potential discomfort during surgery, emphasizing the need for patient cooperation. The patient provided written informed consent.

The patient was admitted 4 days before surgery and fasted during this period. A nasogastric tube was inserted 2 days before surgery for gastric decompression. The anesthetic plan included (1) CSEA under sedation with dexmedetomidine, (2) gastrojejunostomy bypass under spinal anesthesia alone if the epidural catheter was incomplete, (3) dopamine administration to maintain cardiac output, (4) conversion to GA if intraoperative cardiorespiratory instability and/or inadequate sedation developed, (5) chest drain tube insertion if pneumothorax occurred, and (6) intensive care unit (ICU) admission after surgery.

**FIGURE 1:**
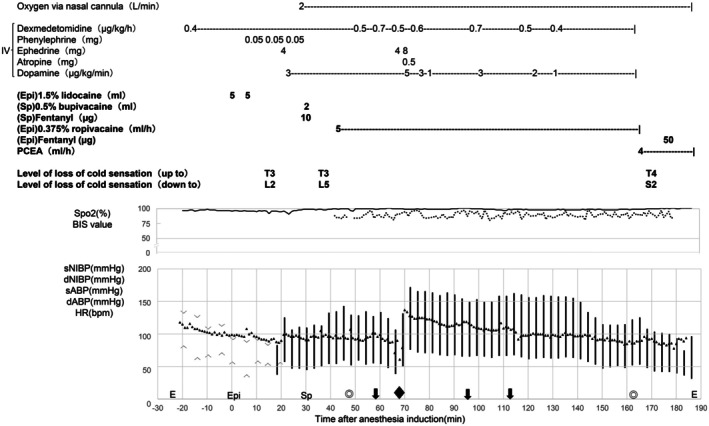
Time after epidural anesthesia induction (min). ―: SpO2, ‐‐‐: BIS value, ▲: HR, ∨: sNIBP (mmHg), ∧: dNIBP (mmHg), |: sABP/dABP (mmHg), E: Operation room enter/exit, ◎: Surgery start/end, Epi: Epidural anesthesia (start of anesthetic administration), Sp: Spinal anesthesia (start of anesthetic administration), ⬇: Event (discomfort and retching), ◆: Event (decreased HR). IV, intravenous; SpO2, percutaneous oxygen saturation; BIS, bispectral index; sNIBP, systolic non‐invasive blood pressure; dNIBP, diastolic non‐invasive blood pressure; sABP, systolic arterial blood pressure; dABP, diastolic arterial blood pressure; HR, heart rate; PCEA, patient‐controlled epidural analgesia (0.2% ropivacaine 280 mL + fentanyl 1000 μg, 4 mL/h).

The patient experienced no nausea or pain upon entering the operating room. Standard monitoring was performed (Figure 1). Dexmedetomidine was started at 0.4 μg/kg/h. An epidural puncture was performed at T7–T8 intervertebral space. An epidural catheter was placed 5 cm cephalad. After confirming correct catheter placement with a 3 mL test dose of 1% lidocaine with adrenaline (1:100,000), 5 mL of 1.5% lidocaine was administered twice. Fifteen minutes later, we confirmed cold sensory blockade in the T3–L2 spinal nerve using an ice pack. Then, we performed spinal puncture at the L3–L4 intervertebral space and injected 2 mL of 0.5% hyperbaric bupivacaine with 10 μg of fentanyl intrathecally, which provided sensory block from T3 to L5. During CSEA induction, phenylephrine was administered to treat hypotension. Since its effect was insufficient, ephedrine was added. In addition, a continuous intravenous dopamine infusion was initiated to maintain stable hemodynamics. The patient received oxygen via a nasal cannula at 2 L/min.

Five minutes after commencing continuous epidural infusion of 0.375% ropivacaine at a rate of 5 mL/h, the operation began with a midline laparotomy extending from the xiphoid process to the umbilicus. Initially, the patient reported no pain, with eyes closed and a relaxed appearance. The patient was able to communicate with the attending anesthesiologist and surgeons. However, when traction was applied to the upper part of the stomach, the patient opened his eyes, complained of mild discomfort, and retched. This event occurred three times during surgery; however, the patient did not vomit. The gastric contents were suctioned using a nasogastric tube. To alleviate the discomfort, we increased the dose of dexmedetomidine, which resulted in hypotension and bradycardia. These side effects were managed with ephedrine, dopamine, and atropine.

Throughout surgery, the bispectral index value was maintained at 80–90s. No intravenous analgesics were required, and no desaturation or breathing difficulties were observed. The abdominal muscles remained relaxed. Abdominal wall closure was performed without difficulty. Surgery (open distal gastrectomy, D1 dissection, and Roux‐en‐Y reconstruction) was performed without any serious complications. The operative time was 115 min. Patient‐controlled epidural analgesia (PCEA) was started (0.2% ropivacaine 280 mL + fentanyl 1000 μg, 4 mL/h), and dexmedetomidine administration was stopped at the end of surgery. Five minutes after the surgery, the patient reported abdominal pain. At that time, the sensory block level was T4–S2. Pain relief was achieved following the administration of 50 μg fentanyl and a single bolus of PCEA.

The patient was transferred to the ICU postoperatively. Fifteen minutes after ICU admission, the patient's blood pressure gradually decreased. PCEA was then temporarily paused. Fluids and noradrenaline were administered. During the ICU stay, he breathed comfortably without requiring respiratory support. Effective postoperative analgesia without nausea or vomiting was achieved using PCEA. The patient was transferred to the surgical ward on postoperative day (POD)1 and was discharged from our hospital on POD12. The patient returned to daily activities without any serious complications and was undergoing outpatient chemotherapy at the time of writing this report (9 months after surgery).

## 3 Discussion

We successfully managed a patient with severe COPD who underwent gastrectomy for gastric cancer, using CSEA with dexmedetomidine sedation. The patient had several PPC risk factors, including severe COPD, upper abdominal surgery, advanced age, and surgery time > 2 h [7, 8, 9]. GA was expected to further increase the risk of PPCs [8, 9]. GA often impairs spontaneous ventilation [10], decreases esophageal sphincter tone, and attenuates protective laryngeal reflexes [11]. During surgery, securing the airway is essential to ensure adequate oxygenation and ventilation and to prevent aspiration. Endotracheal intubation increases the risk of bronchospasm in patients with COPD [12]. A laryngeal mask airway does not reliably protect the lungs from regurgitated stomach contents during gastric surgery [13]. Additionally, in patients with COPD, positive‐pressure ventilation increases air trapping, leading to hypotension, hypercapnia, acidosis, and pulmonary baro‐and volutrauma [12, 14]. Therefore, although gastrectomies are usually performed under GA, we performed RA to preserve spontaneous ventilation and maintain protective laryngeal reflexes without endotracheal intubation.

The combination of GA with RA, rather than GA without RA, promotes postoperative pulmonary function recovery, reduces PPCs, and improves mortality in patients with COPD undergoing major surgery [15, 16]. Additionally, RA, when used as the primary anesthetic modality, decreases PPCs compared with GA in patients with COPD [3, 4]. To our knowledge, no large‐scale studies have directly compared GA alone with RA alone in patients with COPD. RA alone has been widely used in lower abdominal surgery and cholecystectomy to avoid GA [17, 18]. However, reports on its use in gastrectomy for gastric cancer have been extremely limited.

We reviewed reports of gastrectomy performed under RA in patients with respiratory function indicated by an FEV1/FVC ratio of < 0.7 (Table 1) [19, 20, 21]. Cases 1 and 3 received midazolam premedication, whereas Case 3 additionally received a continuous low‐dose remifentanil infusion intraoperatively [19]. In Case 2, the authors suggested that placing the patient in the reverse Trendelenburg position (30°–45°) during the operation might improve breathing [20]. All patients breathed spontaneously throughout the operation without conversion to GA. Although these reports primarily described the intraoperative course, they did not describe survival after discharge. Our patient was the oldest and had the lowest FEV1/FVC ratio among the reported cases, yet was still alive at the time of writing 9 months after surgery. Among these cases, ours was the only case of gastrectomy for diagnosed gastric cancer in which follow‐up after discharge was described.

**TABLE 1:** Summaries of previous reports on gastric surgery with neuraxial anesthesia.

Case	Age	Diagnosis	Comorbidities	FEV1/FVC	Surgery	Anesthesia	Outcome
	Sex						
1 [19]	79	—	Obesity, HT, COPD	0.60	Subtotal gastrectomy	TEA	9‐days hospitalization and discharge
	M						
2 [20]	60	Obesity	COPD, asthma	0.59	Sleeve gastrectomy	TEA	Discharge from hospital on POD3
	M						
3 [19]	75	—	Obesity, HT, Af, DCM, PH, COPD, LC, RA, deafness, AD	0.42	Subtotal gastrectomy	TEA	9‐days hospitalization and discharge
	M						
4 [21]	70	Gastric cancer	COPD, CHF	0.39	Atypical gastric resection	LSA and TEA	Discharge from ICU on POD1
	M						
5 (present case)	80	Gastric cancer	COPD, BPH	0.33	Distal gastrectomy	LSA and TEA	Discharge from hospital on POD12 alive 9th month after surgery
	M						

Abbreviations: AD, anxiety disorder; Af, atrial fibrillation; BPH, benign prostatic hypertrophy; CHF, chronic heart failure; COPD, chronic obstructive pulmonary disease; DCM, dilated cardiomyopathy; FEV1, forced expiratory volume in one second; FVC, forced vital capacity; HT, hypertension; ICU, intensive care unit; LC, lung cancer; LSA, lumbar spinal anesthesia; PH, pulmonary hypertension; POD, postoperative day; RA, rheumatoid arthritis; TEA, thoracic epidural anesthesia.

Additionally, we had waited for 15 min until epidural anesthesia was adequately spread and then assessed the sensory block level before the spinal puncture. When performing CSEA, anesthesiologists generally do not assess the sensory block level of epidural anesthesia until the effects of the spinal anesthesia wear off. Epidural anesthesia fails in up to 30% of clinical cases [22]. If epidural anesthesia is ineffective or insufficient during surgery, conversion to GA may become necessary, or only a gastrojejunostomy bypass procedure may be performed. In the present case, we confirmed that the level of sensory block with epidural anesthesia alone extended from T3 to L2.

Spinal anesthesia was induced in addition to epidural anesthesia in the present case. We assessed the loss of cold sensation using an ice pack to confirm the effectiveness of epidural anesthesia. Since this method may fail to detect patchy blocks, we added spinal anesthesia to supplement any regions that might not have been adequately covered by epidural anesthesia alone.

High spinal anesthesia can cause hypotension and nausea. Sensory block above T4 may induce bradycardia and low cardiac output [23, 24]. Furthermore, relaxation of the intercostal muscles, which are more heavily used in patients with severe COPD, can decrease vital capacity and cause dyspnea [23, 25]. We administered the epidural anesthetic in two divided doses rather than a single bolus, along with a continuous intravenous dopamine infusion, to avoid serious hemodynamic instability and excessive relaxation of the intercostal muscles. Consequently, the patient's hemodynamics remained stable overall, and neither hypotension‐induced nausea nor dyspnea was observed.

We performed sedation in addition to CSEA because visceral discomfort and shoulder pain can arise via the vagus and phrenic nerves, respectively, even with adequate CSEA [26, 27]. Dexmedetomidine, a selective α2‐adrenoreceptor agonist, has sedative, anxiolytic, and analgesic effects with minimal respiratory depression [28]. Dexmedetomidine provided adequate sedation without respiratory depression in the present case, except when traction was applied to the upper stomach. Hypotension and bradycardia, the side effects of dexmedetomidine, were controlled with ephedrine, dopamine, and atropine.

## 4 Conclusion

This report suggests that CSEA with dexmedetomidine may be an effective anesthetic management strategy for older patients with severe COPD undergoing open distal gastrectomy for gastric cancer.

## Author Contributions

**Hiromi Shinohara:** writing – original draft, writing – review and editing. **Gotaro Shirakami:** writing – review and editing. **Maki Ishii:** writing – review and editing. **Masahiro Kakuyama:** writing – review and editing.

## Ethics Statement

The authors have nothing to report.

## Consent

Written informed consent was obtained from the patient for the publication of this case.

## Conflicts of Interest

The authors declare no conflicts of interest.

## Data Availability

The datasets are available from the corresponding author upon reasonable request.
